# 

**Published:** 1908-05-30

**Authors:** 


### BOOKS RECEIVED.

W. Hodge and Co., Glasgow and Edinburgh.

"How to Nurse the Patient." Edited by J. Cowan Wilson, M.B.

Hazell, Watson and Viney, Ltd.

"Mediterranean Winter Resorts." By Eustace Reynolds-Ball, F.R.G.S.

Longmans, Green and Co.

"Quain's Elements of Anatomy." Vol I. Embryology. By T. H. Bryce, M.A., M.D. 11th edition.

J. Murray.

"The Licensed Trade." By Edwin A. Pratt.

### Professor von Noorden on Alcohol.

The misreporting in the Vienna press of some recent remarks by Professor von Noorden upon the use of alcohol in disease, and especially in relation to gout, has induced the speaker to define exactly his views upon the use and abuse of alcohol. Writing to the *Neue Freie Presse*, as translated by the *Times* correspondent, he says: "In regard to gout, I said that persons of gouty disposition must avoid alcohol in every form and quantity, and that I must insist the more upon this inasmuch as I cannot in other respects share the view that alcohol, even in moderate quantities, must be banished from the sick-room and from the table of healthy people. I did not need to demonstrate to my expert audience that alcohol in large quantities is absolutely harmful; but from a scientific standpoint the view is not tenable that small and moderate quantities of alcoholic beverages must be condemned as contributing to individual and racial degeneration. The converse question might be raised whether individuals who cannot stand even the smallest quantity of alcohol ought not to be considered already degenerate. What I said in the clinical lecture room on this purely scientific question was naturally not directed against the highly important and salutary endeavours to combat the abuse of alcohol. Though I have not always been able to approve of the means adopted by the agitators against alcohol, I have always been one of the most zealous opponents of its abuse."

### THE GRADUATES' MEDICAL SCHOOLS.

DIARY FOR WEEK JUNE 1 to 6.

**WEST LONDON HOSPITAL, Hammersmith Road.**

**At 12 noon.**
**June 1,** Dr. Low, **Pathological Demonstration.**
**At 12.15 p.m.**
**June 3** and **5,** Dr. Pritchard, **Practical Medicine.**
**At 5 p.m.**
**June 1,** Mr. Dunn, **Cases of Eye Disease.**
**June 2,** Dr. Low, **Plague.**
**June 3,** Dr. R. H. Cole, **Epilepsy and Insanity.**
**June 4,** Mr. Edwards, **Clinical.**
**June 5,** Mr. Bidwell, **Results after Gastroenterostomy.**

**THE THROAT HOSPITAL, Golden Square, W.**

**At 5.30 p.m.,**
**June 1,** Mr. Rose, **The Accessory Sinuses: Acute Inflammation; Mucoceles.**
**June 4,** Mr. Rose, **The Accessory Sinuses: Chronic Suppuration.**

**CHARING CROSS HOSPITAL, W.C.**
Post-Graduate Demonstrations.

**At 4 p.m.**
**June 4,** Mr. Gibbs, **Surgical.**

**NATIONAL HOSPITAL FOR THE PARALYSED AND EPILEPTIC, Queen Square, Bloomsbury.**

**At 3.30 p.m.**
**June 2,** Dr. Ormerod, **Hysteria.**
**June 5,** Mr. Marcus Gunn, **Optic Atrophy.**

**MOUNT VERNON HOSPITAL, Fitzroy Square.**

**At 5 p.m.**
**June 4,** Dr. T. N. Kelynack, **A Comparative Study of British and Foreign Methods of Combating Consumption.**

**MEDICAL GRADUATES' COLLEGE AND POLYCLINIC, Chenies Street, W.C.**

**At 5.15 p.m.**
**June 1,** Dr. Dundas Grant, **Practical Hints on the Examination of the Nose.**
**June 2,** Mr. J. Cunning, **Some Surgical Affections of the Colon.**
**June 3,** Dr. F. J. McCann, **How to Diagnose Cancer of the Womb.**
**June 4,** Mr. J. W. T. Walker, **The Treatment of Stricture of the Urethra.**

**NORTH-EAST LONDON POST-GRADUATE COLLEGE, Prince of Wales's General Hospital, Tottenham, N.**

**At 4.30 p.m.**
**June 2,** Mr. Carson, **Demonstration of Surgical Cases.**

**THE HOSPITAL FOR SICK CHILDREN, Great Ormond Street, W.C.**

**At 4 p.m.**

**June 1,** Dr. Still, **Mentally Defective Children.**

**LONDON SCHOOL OF CLINICAL MEDICINE, Seamen's Hospital, Greenwich, S.E.**

**At 3.15 p.m.**
**June 1,** Mr. Wm. Turner, **Whitlows.**
**At 2.15 p.m.**
**June 2,** Dr. Hewlett, **Notes on Disinfection and the Use of Disinfecting Agents.**

### Medical Examination of Engine Drivers.

In the House of Commons on May 21 Mr. Lonsdale asked the President of the Board of Trade what results had attended the representations made to railway companies on the subject of the medical examination of engine drivers, following Colonel Yorke's report upon the accident near Atherton. Mr. Churchill said the companies informed him that, generally speaking, enginemen were medically examined on entering the service, after an injury, and in some cases where a superior officer thought re-examination desirable. Recognising the importance of the subject, the companies would continue to give attention to it. The question being difficult, the Board of Trade was bound to consider the conflicting interests and points of view involved. Mr. Macneill inquired whether the President of the Board of Trade had noticed the recent case of an engine driver dropping dead from heart disease. Mr. Hudson wished to know whether the President of the Board of Trade was aware of the irritating conditions from which the men suffered in connection with the medical examination, and whether he would consider the advisability of any medical standard for them being under the authority of the Board itself. Mr. Churchill replied that he would not be justified in making any new departure until the whole subject had been fully considered.

Up to the present time five claims, amounting in all to £84, have been paid by the Employers' Liability Assurance Corporation under "The Doctor's Own Policy." Three of these claims were in respect of cycling accidents, and the other two of measles and diphtheria respectively.

THE HOSPITAL *May* 30, 1908.

*Name* ........................................

*Address* ........................................

........................................

This Coupon must accompany manuscript or contributions intended for The Hospital.

